# Low vitamin D concentrations and BMI are causal factors for primary biliary cholangitis: A mendelian randomization study

**DOI:** 10.3389/fimmu.2022.1055953

**Published:** 2022-12-20

**Authors:** Honglin Xu, Ziyan Wu, Futai Feng, Yongzhe Li, Shulan Zhang

**Affiliations:** ^1^ Department of Rheumatology and Clinical Immunology, Peking Union Medical College Hospital, Chinese Academy of Medical Sciences and Peking Union Medical College, National Clinical Research Center for Dermatologic and Immunologic Diseases (NCRC-DID), Ministry of Science and Technology, Key Laboratory of Rheumatology and Clinical Immunology, Ministry of Education, Beijing, China; ^2^ Department of Clinical Laboratory, Peking Union Medical College Hospital, Chinese Academy of Medical Sciences and Peking Union Medical College, Beijing, China

**Keywords:** primary biliary cholangitis, mendelian randomization, genome-wide association study, body mass index, vitamin D

## Abstract

**Backgrounds:**

Observational studies have identified associations between smoking, alcohol use, body mass index (BMI), and the levels of vitamin D with primary biliary cholangitis (PBC). However, there was a lack of randomization control studies to estimate the causal relationship. This study was to investigate the causal estimates for the effects of those risk factors on PBC.

**Methods:**

The genetic instrument variants were extracted from genome-wide association studies in European ancestry. Two-sample mendelian randomization (MR) and multivariable mendelian randomization were used to determine genetically causal estimates. Primary analyses consisted of random-effects and fix-mode inverse-variance-weighted methods, followed by secondary sensitivity analyses to verify the results.

**Results:**

Our study showed that BMI was a causal factor for PBC (OR 1.35; 95% CI=1.03-1.77; *p*=0.029). In addition, we found that serum vitamin D levels had a protective effect on PBC after adjusting for BMI (OR 0.51; 95% CI=0.32-0.84; *p*=0.007). However, we failed to identify evidence supporting that genetic causal effect of smoking and alcohol intake were associated with PBC in European countries.

**Conclusion:**

Our results enriched findings from previous epidemiology studies and provided evidence from MR that serum vitamin D concentrations and BMI were independent causal factors for PBC, suggesting that ensuing vitamin D sufficiency and healthy lifestyles might be a cost-effective measure for early intervention for PBC.

## 1 Introduction

Primary biliary cholangitis (PBC), formally named primary biliary cirrhosis, was the most common autoimmune liver disease characterized by a chronic immune-driven injury to the small bile duct (1–3). The contemporary prevalence per 100 000 ranged from 1.91 to 40.2 for PBC across Europe, North America, and the Asia-Pacific region (4). While PBC was rare, the clinical burden was disproportionately high relative to population incidence and prevalence. Unfortunately, the etiology of PBC remained largely unresolved, owing to the complexity of the interaction between environmental triggers and genetic susceptibility factors (5).

Epidemiology studies had identified several risk factors as triggers of autoimmune responses in PBC, including smoking, alcohol intake, higher BMI, and vitamin D deficiency although the causality has not been established (6–8). Smoking was identified as an independent risk factor of advanced fibrosis in a French cohort (9), whereas studies from Greece and the Netherlands failed to identify such an association (10, 11). Interestingly, a population-based case-control study showed that alcohol consumption lowered the risk of autoimmune hepatitis, suggesting that alcohol might be a protective factor for the development of PBC (12). Furthermore, increasing evidence suggested a nonskeletal role of serum 25-hydroxyvitamin D [25(OH)D] levels in PBC (13–15). The most recent observational evidence showed serum 25(OH)D levels were associated with incomplete response to ursodeoxycholic acid (UDCA), cirrhosis development, and clinical outcomes in patients with PBC (16). Given these inconsistent findings and absence of evidence from randomized controlled trials, it remains controversial whether the causal effects exist between these risk factors and the development of PBC.

Multivariable mendelian randomization (MVMR) is a form of instrumental variable (IV) analysis that uses genetic variants, normally single nucleotide polymorphisms (SNPs), as instruments to obtain estimates of the direct causal effect of each exposure included in the estimation on the outcomes (17). It is a comparable method with randomized control trials that free from bias due to unobserved confounding, measurement errors and reverse causations (18). This method, together with the wide availability of published genome-wide association study (GWAS) summary datasets, potentiates mendelian randomization (MR) a time- and cost-efficient approach and contributes to its increasing popularity for assessing and screening for potentially causal associations (19). Further, selecting genetic variants located in genes with biological function might provide an opportunity to clarify causal mechanisms (20). To systemically investigate the causal effects of smoking, alcohol, BMI, and serum 25(OH)D levels on the risk of PBC, we conducted a two-sample MR study. To simultaneously investigate the independent effect of each risk factor, we performed a multivariable mendelian randomization study.

## 2 Methods

### 2.1 Exposure and outcome data sources

All of the summary datasets were obtained from the MRC IEU Open GWAS database (https://gwas.mrcieu.ac.uk/). PBC GWAS dataset consists of 2764 cases and 10475 controls from European populations. All PBC cases included in the cohorts fulfilled the American Association for the Study of Liver Diseases criteria for PBC (21). Summary statistics data for smoking initiation and drinks per week were available at the GWAS and Sequencing Consortium of Alcohol and Nicotine use (GSCAN), which consists of 607,291 samples and 335,394 samples, respectively (22). Summary datasets for serum 25(OH)D levels were obtained from GWAS based on large UK Biobank (UKB) consisting of 417,580 Europeans. Distribution of 25(OH)D concentrations in the UK Biobank sample showed seasonal fluctuation with median, mean and interquartile ranges of 47.9, 49.6, 33.5–63.2 nmol L^−1^. Levels of serum vitamin D deficiency were defined as below 25 nmol L^−1^ (23). Summary statistics of BMI were obtained from MRC-IEU in 461,460 samples (IEU GWAS ID: ukb-b-19553). To eliminate population stratification bias, all summary data were retrieved from studies that solely included populations of European ancestry.

### 2.2 Selection of instrumental variables

All genetic variants achieving genome-wide significance were selected as instrumental variables (IVs) at the *p*-value cutoff of 5 × 10^-8^. To guarantee the variants were independent, SNPs in high linkage disequilibrium (r^2^ >0.001 or clump windows<10,000kb) were excluded. To prevent the effect estimates from aligning with different alleles, harmonization was performed to remove ambiguous SNPs showing non-concordant alleles. The maximum minor allele frequency (MAF) threshold for aligning palindromic SNPs was set for 0.01. When an exposure-associated SNP was not present in the outcome dataset, a proxy SNP, which is highly correlated with the variant of interest (r^2^ > 0.8) was selected instead. We utilized these carefully chosen SNPs as the final genetic IVs for the subsequent MR analysis.

F statistic for each SNP was calculated using the following formula: Beta^2^/SE^2^. Beta and SE denote the estimate and standard error of effect allele on exposures. The proportion of variance explained by each SNP was calculated using the following formula: 2 × Beta^2^ × MAF × (1–MAF) (24). If F>10, the correlations between the IVs and exposure were considered sufficiently strong, thus the results of the MR analysis could avoid being affected by weak-tool bias (25).

### 2.3 Statistical analysis

The inverse variance weighted (IVW) method was selected as the main two-sample MR analytical method to estimate the causal effect (26). MR-Egger regression, the Weighted Median, and the Weighted Mode methods, and MR-pleiotropy adjusted profile score (MR-PRESSO) were used to infer the causal relationship. The causal effect of exposure on PBC was considered indicative if the effect estimate was significant in the IVW method and no contradictory results were found in other methods.

Various methods were introduced in this study for pleiotropy and heterogeneity analysis. Firstly, the heterogeneity of IVs was assessed *via* Cochran’s Q test. If significant heterogeneity was detected in some exposures, the random effect model was used to estimate the MR effect size; otherwise, the fixed-effects IVW method was considered the main result. Secondly, potential horizontal pleiotropy of IVs was evaluated by the MR-Egger regression intercept. If the *p*-value was less than 0.05, the MR analysis might obey the hypothesis that genetic exposure influenced the outcome directly (27). Thirdly, leave-one-out sensitivity test examined whether a single SNP caused the results. Additionally, we applied MR pleiotropy residual sum and outlier test (MR-PRESSO) to detect potential horizontal pleiotropy using the MR-PRESSO global test. Leave-one-out analysis to determine whether outliers may be biasing the overall MR estimate. For any detected pleiotropic SNP, the MR-PRESSO outlier test was performed to remove these SNPs and rectify the horizontal pleiotropy (28). Finally, funnel plots and scatter plots were evaluated as a visual inspection of symmetry and the effect estimates. To account for multiple testing in our analyses of 4 exposures in relation to PBC, we used a Bonferroni-corrected threshold of p value = 0.0125 (*α* = 0.05/4 risk factors). Associations with *p* -values between 0.0125 and 0.05 were considered suggestive evidence of associations, requiring confirmation. All tests were two-sided and performed using the “TwoSampleMR”, “MR-PRESSO” packages in the R software (version 4.0.2).

### 2.4 Multivariable mendelian randomization

We next conducted multivariable MR analysis which included SNPs that were genome-wide significant in alcohol intake and smoking initiation, serum 25(OH)D levels, and BMI. The effects of genetically predicted risk factors were estimated using the function mv_multiple in “TwoSampleMR” package.

### 2.5 Ethical approval

Our study only involved the collection or study of existing data and documents which were obtained from published studies approved by the corresponding ethics committee, thus no further ethical approval was required for this study.

## 3 Results

### 3.1 Univariable mendelian randomization analysis

**Figure 1:**
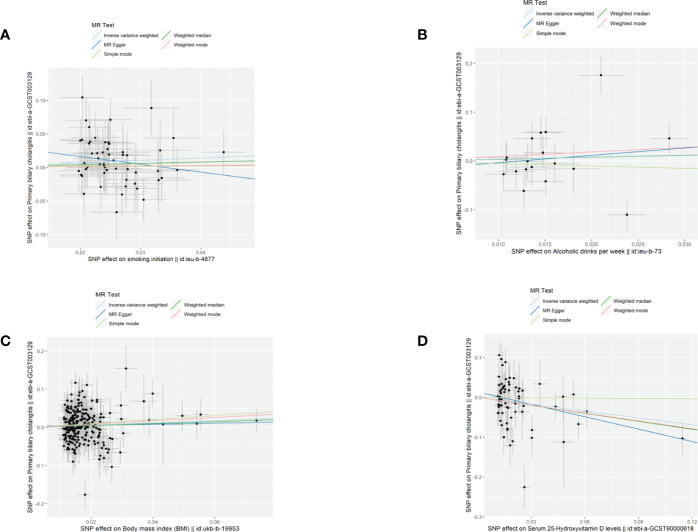
Scatter plots demonstrated causal effects in univariable MR of PBC. **(A)** smoking initiation; **(B)** drinks per week; **(C)** body mass index, BMI; **(D)** serum 25-Hydroxyvitamin D levels.

A total of 69 SNPs that were associated with serum 25(OH)D levels were extracted. Univariable MR showed that genetically predicted serum 25(OH)D levels were negatively associated with PBC except for the simple mode method (**Figure 1D**). The F-statistics of IVs ranged from 29.94-1474.55, indicating robust correlations between IVs and serum vitamin D levels(**Supplementary Table 1**, **Table 1**). The odds ratio (OR) was 0.56 (95%CI: 0.51-0.93, *p*=0.020) per one standard deviation (SD) increase in the levels of serum 25(OH)D by IVW fixed-mode method, which was consistent with multiplicative random mode IVW method. MR-PRESSO identified two influential outliers which were rs13108245 and rs142158911 (outlier test *p*-value<0.08). After removing these outliers, the causal effect estimate remained significant (OR 0.60, 95%CI: 0.40-0.89, p=0.014).

**Table 1:** Univariable Mendelian randomization of exposures on the risk for PBC.

Exposure	Consortium	Exposrue N	N.SNPs	method	OR ( 95%CI )	p
Smoking initiation	GSCAN	607,291	55	MR Egger	0.31 (0.05, 2.07)	0.233
				Weighted median	1.22 (0.72, 2.09)	0.459
				IVW (fixed mode effects)	1.43 (0.99, 2.05)	0.055
				Simple mode	1.05 (0.32, 3.47)	0.932
				Weighted mode	1.08 (0.41, 2.84)	0.883
Drinks per week	GSCAN	335,394	16	MR Egger	26.39 (0.46, 1509.69)	0.135
				Weighted median	2.34 (0.48, 11.48)	0.296
				IVW (fixed mode effects)	1.72 (0.54, 5.46)	0.358
				Simple mode	0.63 (0.02, 17.98)	0.793
				Weighted mode	2.92 (0.35, 24.49)	0.339
Body mass index	MRC-IEU	461,460	252	MR Egger	1.14 (0.56, 2.34)	0.718
				Weighted median	1.29 (0.81, 2.05)	0.280
				IVW (fixed mode effects)	1.35 (1.03, 1.77)	0.029
				Simple mode	1.69 (0.57, 5.03)	0.348
				Weighted mode	1.55 (0.86, 2.79)	0.145
				IVW (multiplicative random effects)	1.35 (1.03, 1.77)	0.029
				MR-PRESSO outlier corrected	1.38 (1.09, 1.75)	0.005
Serum 25(OH)D levels	MRC-IEU	496,946	69	MR Egger	0.35 (0.15, 0.81)	0.016
				Weighted median	0.51 (0.29, 0.90)	0.019
				IVW (fixed mode effects)	0.56 (0.35, 0.91)	0.020
				Simple mode	0.97 (0.33, 2.89)	0.958
				Weighted mode	0.52 (0.30, 0.91)	0.024
				IVW (multiplicative random effects)	0.56 (0.35, 0.91)	0.020
				MR-PRESSO outlier corrected	0.60 (0.40, 0.89)	0.014

SNPs, Single nucleotide polymorphisms; OR, Odds ratio; CI, Confidence interval; IVW, inverse-variance weighted; MR-robust adjusted profile score; MR-PRESSO, MR-pleiotropy residual sum and outlier.

A total of 252 SNPs that were associated with BMI were extracted. Univariable MR showed that genetically predicted BMI was associated with PBC by IVW method (**Figure 1C**, **Supplementary Table 2**, **Table 1**). One SD increase in BMI was found to increase PBC risk, with an IVW OR of 1.35 (95% CI: 1.03-1.77, *p*=0.029). However, the casual effect was not significant in other sensitivity analyses, including MR Egger, Weighted median, simple mode, and weighted mode. In the main analysis, MR-PRESSO identified influential outliers which were rs75499503, rs3803286 and rs1064213 (outlier test *p* < 0.304). After removing these outliers, the OR was 1.38 (95%CI: 0.40-0.89, *p*=0.005). The MR-Egger intercept indicated no further directional pleiotropy once these variants were removed (**Supplementary Table 2**).

Genetic predisposition to smoking initiation and alcohol intake was not associated with PBC (**Figures 1A, B**). The IVW OR per unit increase in log odds of smoking initiation was 1.43 (95% CI: 0.99-2.05, *p*=0.055) and 1.72 (95% CI: 0.54-5.46, *p*=0.358) for alcohol intake per week. The effects were consistent across the four MR methods. A total of 55 SNPs were selected as IVs for smoking initiation and 16 SNPs for alcohol intake per week. MR-PRESSO identified rs76640332 and rs1260326 as outliers of alcohol intake per week (outlier test *p*<0.018). The results of IVW (multiplicative random effects) and MR-PRESSO outlier corrected for smoking initiation were not available as no heterogeneity and no outlier was identified. (**Table 1**; **Supplementary Tables 3** and **4** )

### 3.2 Heterogeneity and pleiotropy analysis

In the heterogeneity test, the *p*-values of Cochran’s Q statistics were smaller than 0.05, indicating heterogeneity in the SNPs effect estimates of BMI, serum 25(OH)D levels, and alcohol. In the horizontal pleiotropy test, the MR-Egger regression intercept indicated no evidence of pleiotropy (**Table 2**). Funnel plots indicated heterogeneity. We excluded rs76640332 and rs1260326 that significantly influenced the results in leave-one-out analysis of alcohol intake. The leave-one-out method showed that the potential causal correlation between smoking initiation, alcohol intake per week, BMI, and serum 25(OH)D levels and PBC risk were not driven by a single SNP (**Supplementary Figures 1**–**4**).

**Table 2:** Pleiotropy and heterogeneity test of the four exposures IVs from PBC GWAS.

Exposure	Heterogeneity test						Pleiotropy test		
	MR-Egger			Inverse variance weighted			MR-Egger		
	Q	Q_df	Q_pvalue	Q	Q_df	Q_pvalue	Intercept	se	p
Smoking initiation	47.438	53	0.69	50.009	54	0.629	0.04	0.025	0.115
Drinks per week	13.361	14	0.498	15.263	15	0.433	-0.043	0.031	0.189
Body mass index	312.383	250	0.004	312.687	251	0.005	0.003	0.007	0.622
Serum 25(OH)D levels	114.419	67	2.75E-04	117.582	68	1.80E-04	0.014	0.01	0.178

df, degree of freedom; MR, Mendelian randomization; Q, heterogeneity statistic Q.

### 3.3 Multivariable mendelian randomization results

**Figure 2:**
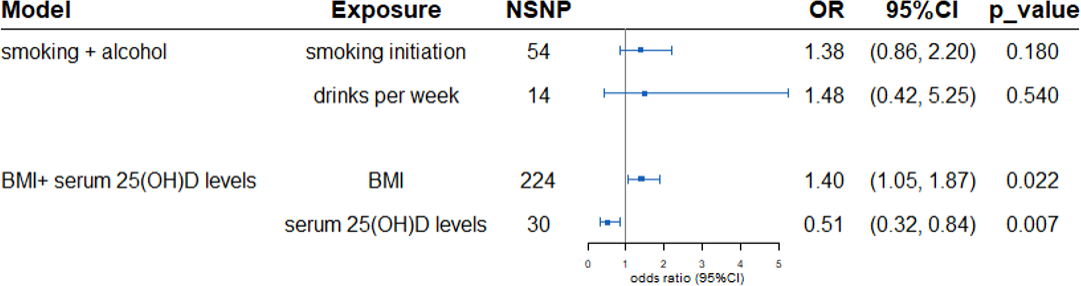
Multivariable Mendelian randomization estimated the association between exposures and outcome PBC adjusts of confounding. In the smoking and drinks model, for alcohol, OR is per 1 SD increase in log-transformed weekly alcohol drinks consumed (multivariable MR adjusts for smoking). For smoking, OR is per 1 SD increase in the log odds of smoking initiation (multivariable MR adjusts for alcohol consumption). Similar in BMI and serum 25(OH)D levels model. SNPs, Single nucleotide polymorphisms; OR, Odds ratio; CI, Confidence interval; BMI, body mass index.

Multivariable MR was utilized to investigate the direct effect of the four exposures on PBC. After adjustment of BMI, the protective effect of serum 25(OH)D levels was further enhanced (OR 0.51, 95%CI: 0.32-0.84, p=0.007). A significant causal effect of BMI on PBC was observed after adjustment of serum 25(OH)D levels. In the multivariable MR analysis mode controlling for alcohol intake per week, there was no causal effect of smoking initiation on risk of PBC (OR 1.48, 95%CI: 0.42-5.25; *p*=0.540). In multivariable MR analysis controlling for smoking, the genetic causal effect of alcohol intake per week remained insignificant (OR 1.38, 95%CI: 0.86-2.20; *p*=0.180) (**Figure 2**).

## 4 Discussion

We used two-sample-MR and multivariable MR to systematically explore genetically causal effects of smoking, alcohol intake per week, BMI, and serum 25(OH)D levels on the risk of PBC. Our study showed that BMI was a causal factor for PBC. Further, we showed that serum 25(OH)D had a protective effect on PBC after adjusting for BMI. However, we did not observe evidence supporting that genetic causal effects of smoking and alcohol intake were associated with PBC in European countries.

It has been shown that the risk of cirrhosis development and liver-related mortality was higher in patients with vitamin D levels below 50 nmol/L (16), which supported our findings that higher vitamin D levels had a protective effect on PBC. Furthermore, a retrospective study demonstrated that vitamin D deficiency at baseline was associated with incomplete response to ursodeoxycholic acid (UDCA), a first-line FDA-approved drug for PBC (13). In consistent with these findings, we demonstrated that, for each genetically determined unit increase in log-transformed VD levels, the risk of PBC was reduced by 44% (OR=0.56), and by 49% after adjusting for BMI. Two major processes, including activation of nonparenchymal liver cells and the excessive deposition of extracellular matrix, are shown to contribute to hepatic fibrogenesis. Vitamin D receptor is not expressed in liver tissue but in nonparenchymal liver cells. Decreased vitamin D-vitamin D receptor/miRNA155-suppressor of cytokine signaling 1 axis may lead to insufficient negative regulation of cytokine signaling and play an important role in the pathogenesis of PBC (29). Additionally, vitamin D suppresses the expression of collagen I and III and enhances the expression of matrix metalloproteinase-8 (MMP8), a member of MMP family responsible for degradation of extracellular matrix (30). Moreover, vitamin D plays an important role in regulating T-cell-mediated immunity as it reversed inflammatory CD28^-^ T cells accumulate in livers of patients with primary sclerosing cholangitis and localize around bile ducts (31). Taken together, these studies corroborate our findings that vitamin D is protective against PBC.

Our results indicated that genetically determined increase in BMI was a causal factor for PBC dependent on serum vitamin D levels. Although the underlying mechanisms of how higher BMI promotes the development of PBC remains unclear, it has been shown that adipose tissue dysfunction is characterized by increased inflammation, impaired extracellular matrix remodeling and fibrosis together with an altered secretion of adipokines. As adipokines easily reach the liver through the portal vein, which may lead to local vascular effects, worsening the portal hypertension (32). Moreover, in high-fat diet mice and PBC murine models, IFNγ signaling is crucial for obesity-mediated inflammatory responses (33). Furthermore, our findings were in consistent with previous observations that PBC patients with BMI≥25 were more likely to develop hepatocellular carcinoma, and were associated with severe biliary duct damage and fibrosis (34, 35). Therefore, additional studies are needed to provide mechanistic evidence of the relationships between obesity and PBC. In addition, it is necessary to monitor the fluctuation of BMI in patients with PBC, especially those complicated with metabolic syndrome, diabetes, and steatosis (7, 36).

There remains a paucity of consistent evidence supporting the pathogenic role of smoking and alcohol. Overall, a meta-analysis showed significantly increased risk of PBC among former smokers with a pooled OR of 1.31 (37). In contrast, studies from Greece (11) and the Netherlands (10) did not identify any associations between smoking and increased risks of PBC. Previous epidemiological studies suggested that mild to moderate alcohol intake was negatively associated with PBC (38, 39). In our study, independent genetic variants, which were strongly associated with smoking and alcohol, were selected as instrumental variables from large GWAS summary data. Surprisingly, we did not observe significant correlations between smoking and alcohol intake and increased PBC risks. Further, multivariable MR failed to identify direct effect of smoking or alcohol intake on PBC after adjusting for confounders. The discrepancies among these findings remain unclear, as smoking history and alcohol consumption history data were collected from PBC patients using a questionnaire, which may subject to recall bias and differential measurement error. Further studies are needed to further verify our findings.

Our study has several strengths. We, for the first time, utilized a multivariable MR design to investigate the causal effects of smoking, alcohol, BMI, and serum 25(OH)D levels on the risk of PBC. Second, we used the multiple sensitivity methods on novel large GWAS datasets, in which the genetic landscape of risk factors in PBC has been greatly expanded. Meanwhile, there are several limitations in our study. The study population included in the PBC cohort was from Canada, Italy and the United Kingdom (**Supplementary Table 5**). Whether the results can be reproducible in other populations remains to be verified. Next, there were significant heterogeneities across genetic variants in serum vitamin D levels and BMI. The heterogeneities may derive from analyses platforms, participants and huge number of SNPs. It was important to note that PBC was an autoimmune disease as a result of complex interactions between genetic and environmental factors and MR studies were purely statistical constructs. Thus, those causal factors should be applied to the clinic carefully.

In conclusion, we enriched findings from previous epidemiology studies and provided evidence from MR that serum 25(OH)D and BMI were independent causal factors for PBC, suggesting that ensuing vitamin D sufficiency and a healthy lifestyle might be a cost-effective measure for early intervention. Further work using updated MR analysis based on larger scale GWAS summary data and more ancestry groups may provide more robust evidence for environmental risk factors on PBC.

## Data availability statement

The datasets presented in this study can be found in online repositories. The names of the repositories and GWAS ID can be found in the article.

## Author contributions

Study design: HX, ZW. Data collection and data analysis: HX, ZW. Data interpretation: HX, ZW, FF. Drafting manuscript: HX, ZW, SZ, YL. All authors take responsibility for the integrity of the data analysis. All authors contributed to the article and approved the submitted version.
