# Supplementary figures and images for: Low vitamin D concentrations and BMI are causal factors for primary biliary cholangitis: A mendelian randomization study
Source: Front Immunol. 2022 Dec 20;13:1055953. doi: 10.3389/fimmu.2022.1055953 (PMC9807903; doi:10.3389/fimmu.2022.1055953)

## Slide 1
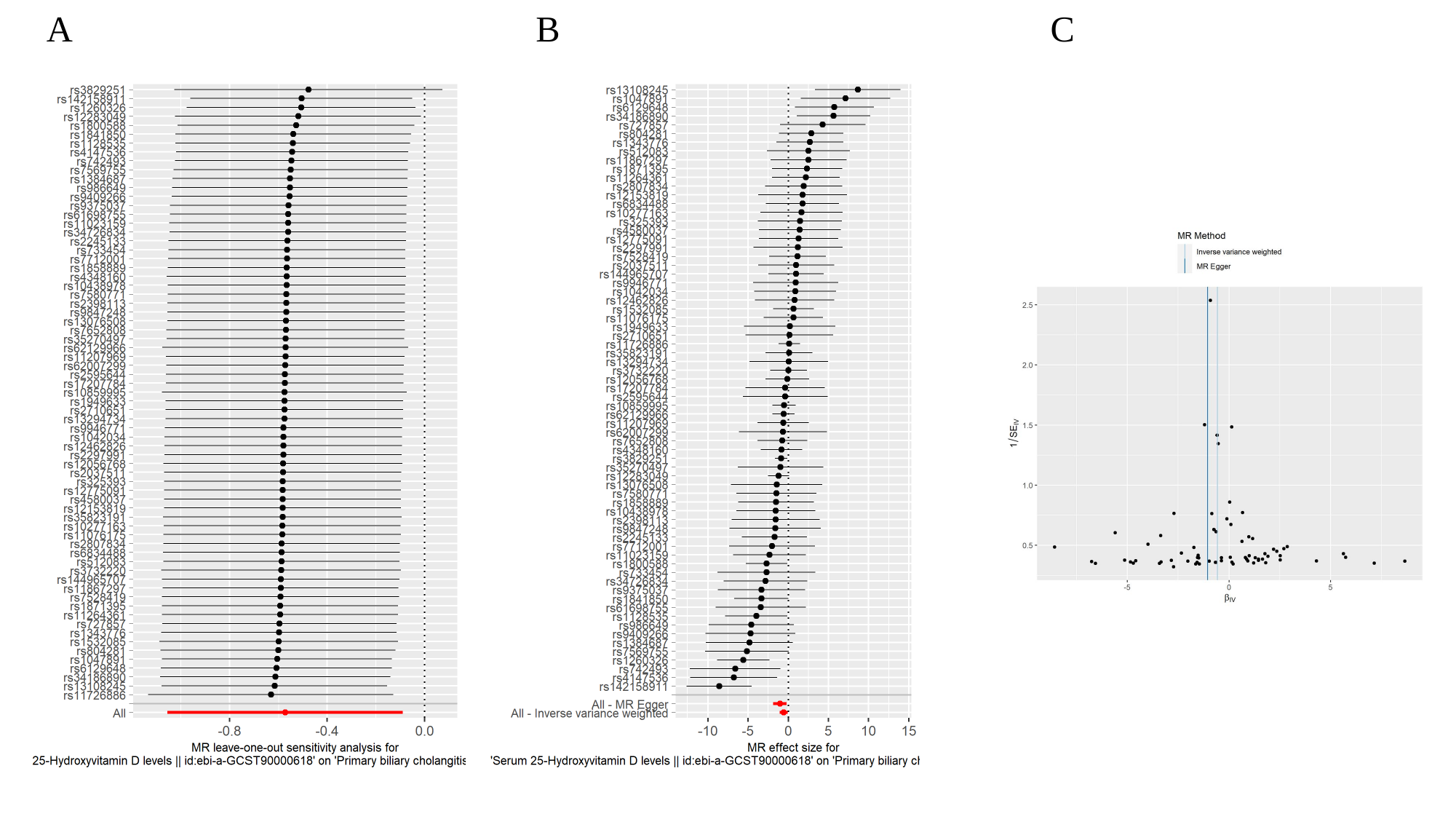

C
A
B

## Slide 2
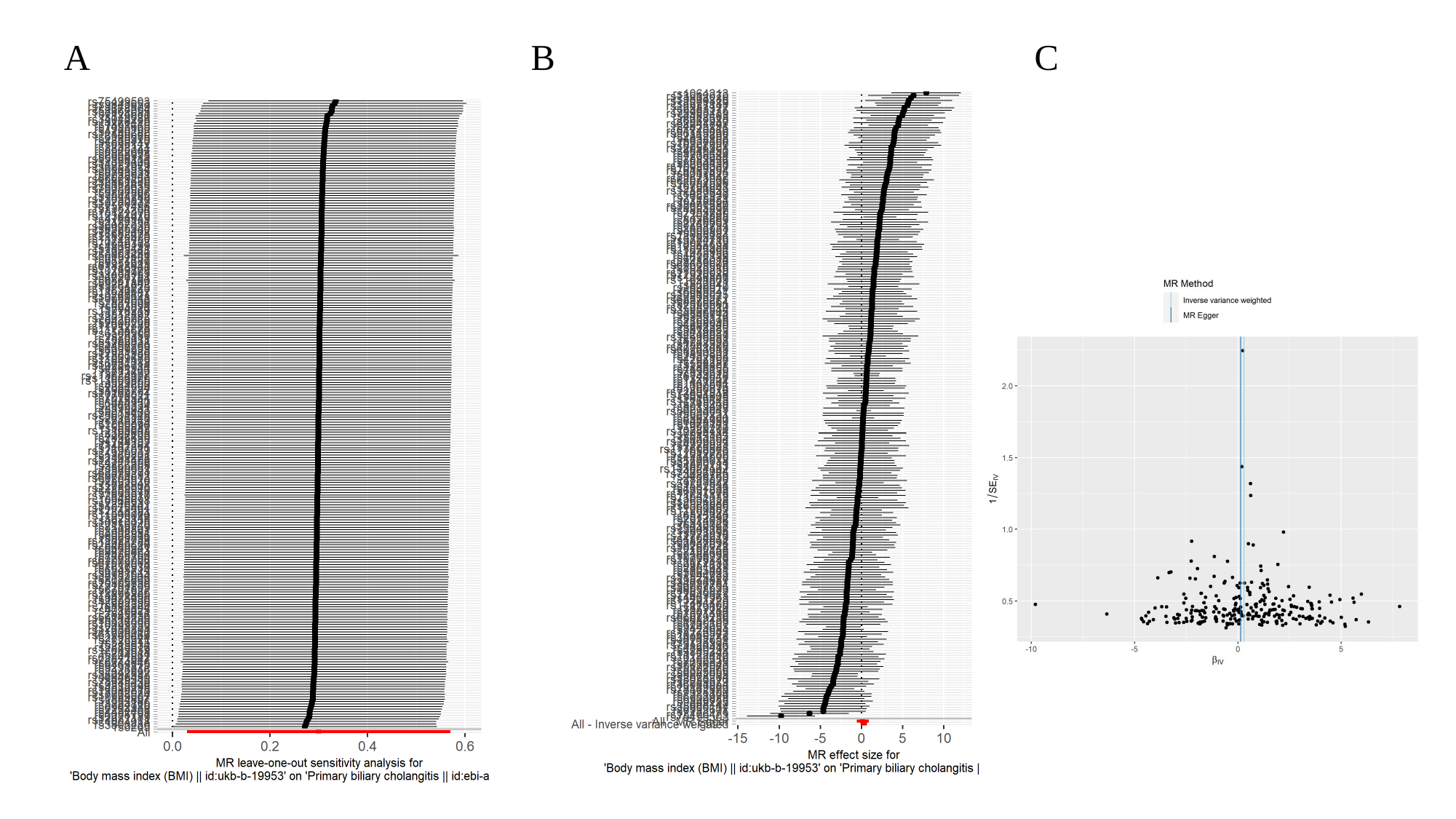

C
B
A

## Slide 3
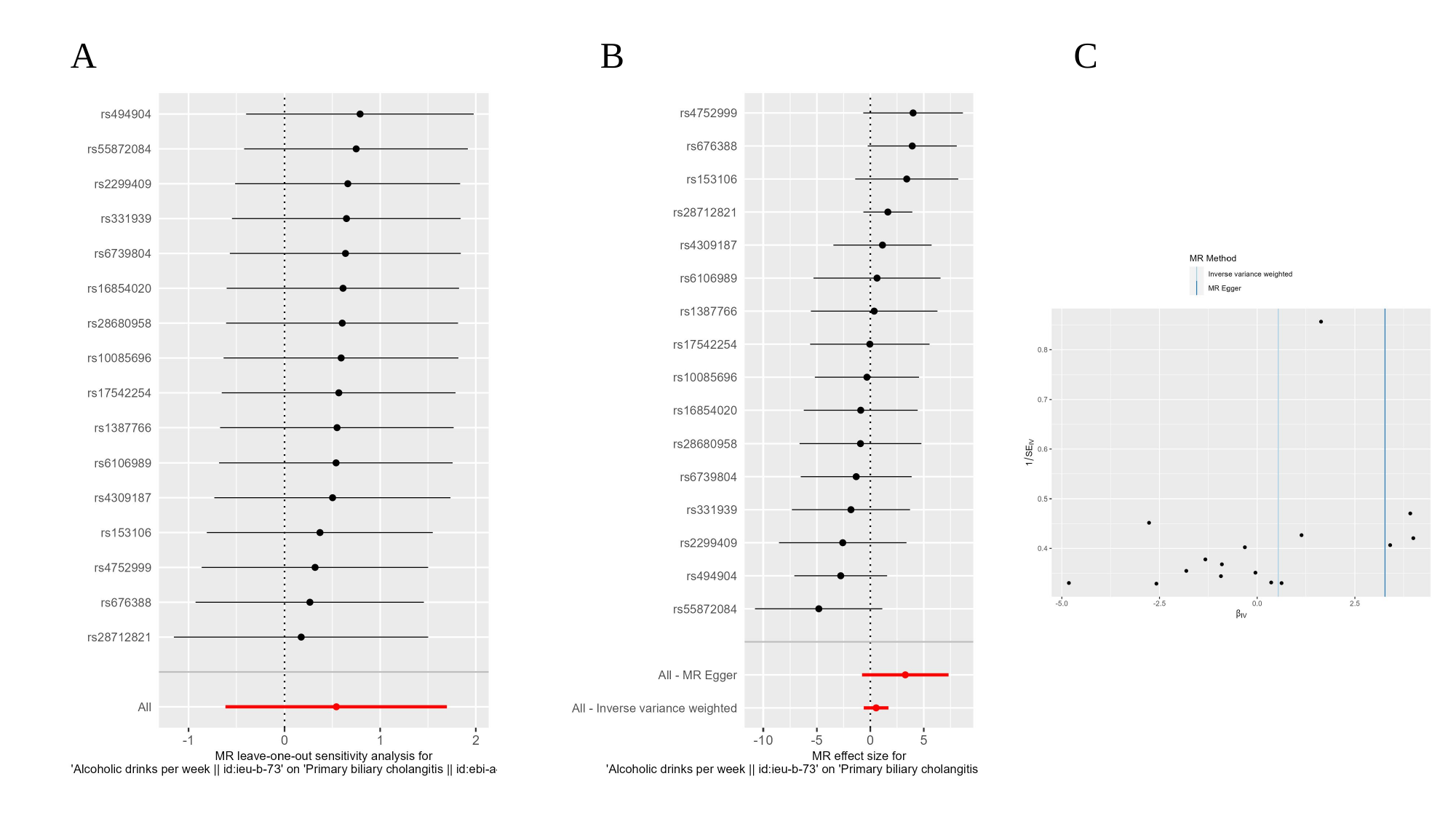

A
C
B

## Slide 4
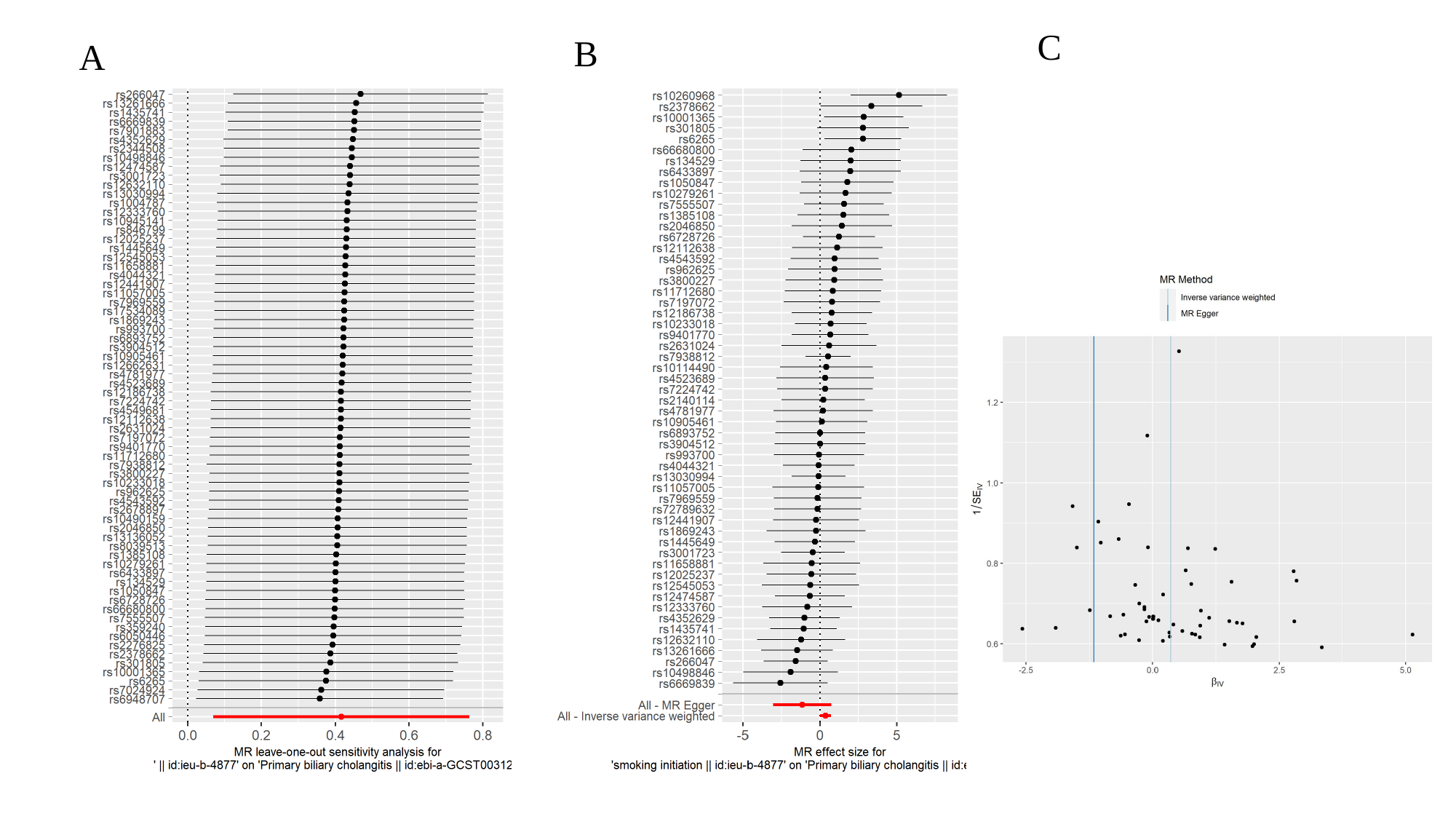

C
B
A

Supplement: Supplementary file 1 [file Presentation_1.pptx]
